# A new species of *Epidendrum* L. (Orchidaceae) of pendulous habit from Peru

**DOI:** 10.3897/phytokeys.184.70844

**Published:** 2021-11-03

**Authors:** Luis Ocupa Horna, Eric Hágsater, Marco M. Jiménez

**Affiliations:** 1 Departamento de Orquideología, Centro de Investigación en Biología Tropical y Conservación-CINBIOTYC, Piura, Perú Centro de Investigación en Biología Tropical y Conservación Piura Peru; 2 Departamento de Orquideología, Instituto de Ciencias Antonio Brack, Lima, Perú Grupo Científico Calaway Dodson: Investigación y Conservación de Orquídeas del Ecuador Quito Ecuador; 3 Grupo Científico Calaway Dodson: Investigación y Conservación de Orquídeas del Ecuador, Quito, 170510, Pichincha, Ecuador Instituto de Ciencias Antonio Brack Lima Peru; 4 Herbario AMO, Montañas Calizas 490, México, CDMX 11000, México Herbario AMO Mexico City Mexico; 5 Vivero de Conservación La Paphinia, Avenida del Ejército y Juan Izquierdo, Zamora, Zamora Chinchipe, 190102, Ecuador Vivero de Conservación La Paphinia Zamora Chinchipe Ecuador

**Keywords:** Cajamarca, endemic, Epidendroideae, epiphyte, neotropic, San Ignacio

## Abstract

A new species of *Epidendrum* L. from northern Peru is described, illustrated, and compared with related species. This new species belongs to the Laxicaule Group and shares morphological characteristics with *Epidendrumlaxicaule* D.E. Benn & Christenson but differs in the shape and length of the dorsal sepal; the shape of the petals and the lip, ribs position of the lip, shape of the vesicle formed between the ovary and the column as well as the section of the stem.

## Abstract (es)

Se describe, ilustra y compara una nueva especie de *Epidendrum* L. del norte de Perú con otras especies relacionadas. Esta nueva especie pertenece al Grupo Laxicaule y comparte características morfológicas con *Epidendrumlaxicaule* D.E. Benn & Christenson pero difiere en la forma y longitud del sépalo dorsal; la forma de los pétalos y del labio, la posición de las costillas del labio, la forma de la vesícula formada entre el ovario y la columna así como la sección del tallo.

## Introduction

The genus *Epidendrum*Linnaeus (1763: 1347) are terrestrial, epiphytic and lithophytic plants that occur in different types of vegetation ranging from tropical forests, coastal dunes and scrubs to Andean paramos (Hágsater and Soto 2005; Chase et al. 2015). The genus ranks as one of the most diverse among Neotropical orchids, with around 2400 species (Hágsater et al. 2016), 1500 of them being recently treated and illustrated in the Icones Orchidacearum series (Hágsater and Salazar 1993; Hágsater et al. 1999; Hágsater and Sánchez 2001, 2004, 2006, 2007, 2008, 2009, 2010, 2013, 2015a, 2015b; Hágsater and Santiago 2018a, 2018b, 2019, 2020a, 2020b, 2021).

Though there have been many attempts to separate *Epidendrum* into various genera (Hágsater 1985; Hágsater and Soto 2005), it has been argued that it is best left as a single large genus reflecting its monophyly, as revealed by molecular studies (Hágsater and Soto 2005; Hágsater et al. 2019; Granados et al. 2020). Groups of species within *Epidendrum* can be cohesively aggregated based on vegetative and floral characteristics, which are also supported by molecular information. As this work is in progress, no formal sub-generic classification has been proposed to date, based on the informal groups recognized in current taxonomic practice (Hágsater 1985; Hágsater and Huayta 2018).

This is the case of the Laxicaule group, endemic to Peru, which is characterized by the pendulous, monopodial habit, with sub-apical branching, the laterally compressed to ancipitous stems, the relatively short leaves, the short racemose, few-flowered inflorescences, the large flowers and the lip with prominent ribs (Hágsater and Huayta 2018). Presently, there are only two species in the group: *Epidendrumlaxicaule* D.E. Benn & Christenson and *E.megalopentadactylum* Hágsater & Huayta (Hágsater and Huayta 2018) found in central Peru. Here we are describing, illustrating and representing with photographs a new species of *Epidendrum*, *E.lufinorum* Ocupa & Hágsater, as well as photographs of the related species and two undescribed species. A table is provided with the characteristics that distinguish the three species of the Laxicaule Group for which information is available.

## Material and methods

A living plant in flower of the new species was collected in March 2016 during a botanical expedition to Cerro Parcos in the department of Cajamarca, northeastern Peru. The photographs were taken in situ using a Canon Rebel T3 camera equipped with a Canon EF-S 18–55mm f/3.5–5.6 lens and were later used for preparing the line drawing and figures. Fresh flowers were preserved in 70% ethanol and 1% glycerol. The single collected plant was dried to make a herbarium specimen, which was afterwards deposited in the Herbarium Truxillense (HUT, acronym following Thiers 2021, updated continuously), Trujillo, Peru.

In order to determine the taxonomic status of the collected specimen, we examined all relevant *Epidendrum* material from USM and MOL (acronym following Thiers 2021, updated continuously), because both herbaria have a large collection of *Epidendrum* species. The original descriptions from holotypes of related species (Bennett and Christenson 1998; Hágsater and Santiago 2018a) were consulted and compared. Additionally, some online resources including the scans of relevant type specimens were accessed such as the JSTOR Global Plants web portal (https://plants.jstor.org). A distribution map of the proposed new species and related species, was prepared using the software QGIS 3.10 (QGIS Development Team 2020). The holotype specimen was collected under the research permit for the project “Estudios Taxonómicos Selectos de la Flora del Norte del Perú” with Resolution N° 247 –2016 –SERFOR/DGGSPFFS and the extension Resol. 430–2017.

## Taxonomic treatment

### Epidendrum lufinorum

Taxon classificationPlantaeAsparagalesOrchidaceae

Ocupa & Hágsater sp. nov.

87EA92D4-77CA-5E82-A084-1188ECD2E11D

urn:lsid:ipni.org:names:77221545-1

Figs 1 , 2 , 4C

#### Type.

Peru. Cajamarca: San Ignacio, in a coffee plantation, close to the caserío Villa Rica, DDM 5°5.1607'S, 78°53.2076'W, elev. 1690 m, 03 April 2016, *Ocupa 211* (holotype: HUT!).

Similar to *Epidendrumlaxicaule* D.E.Benn & Christenson, but differs in having smaller dorsal sepal (i.e. 2.4 × 0.4 cm vs. 3.2 × 0.6 cm) which is oblong-oblanceolate (vs. narrowly oblanceolate), with an obtuse apex (vs. acute), the linear and obtuse petals (vs. narrowly linear-lanceolate and acuminate), lip transversely cordate (vs. transverse), disc with 5 parallel and central ribs (vs. 5 parallel ribs projecting distally), a gibbous vesicle (vs. globose) and the stem section terete (vs. elliptic).

**Figure 1.**
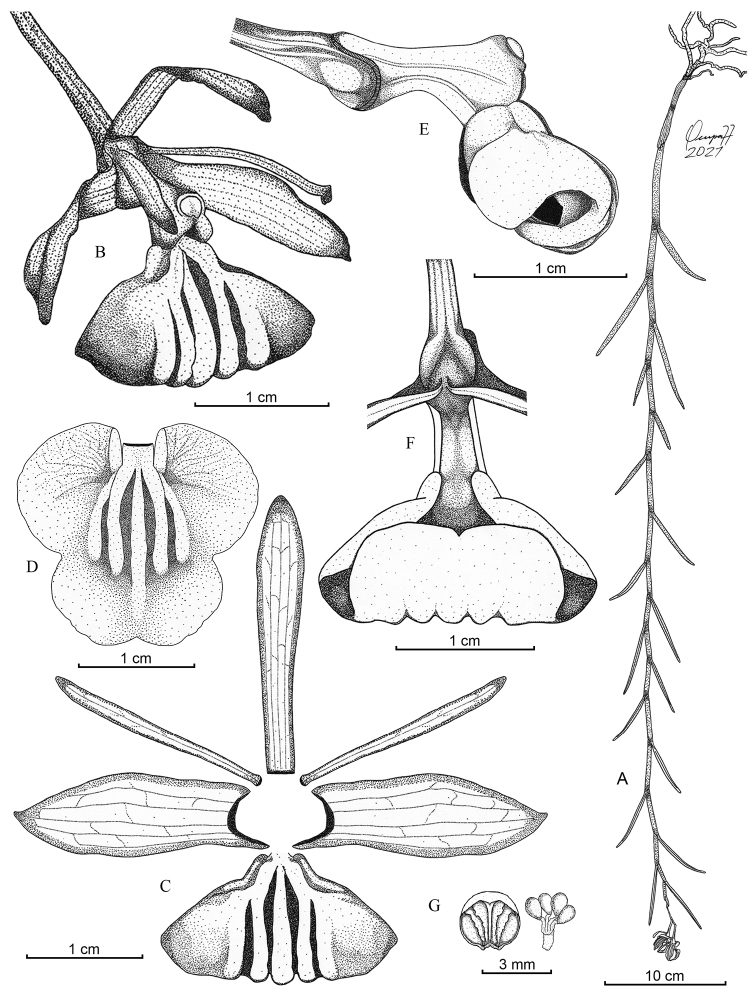
Drawing of *Epidendrumlufinorum* sp. nov. **A** habit **B** flower **C** dissected perianth **D** lip extended **E** column, lip and ovary, lateral view **F** lip in natural position, abaxial view **G** anther and pollinarium. Illustration by L. Ocupa from the holotype (*L. Ocupa* 211, HUT).

#### Description.

Epiphytic ***herb***, monopodial, branching, pendulous, slender, about 72 cm long including inflorescence. ***Roots*** 2 mm in diameter, basal, white, fleshy. ***Stems*** ca. 65 cm long, terete in cross section, new shoots produced from sub-apical nodes of primary stem, enveloped by tubular, fleshy, articulated, green with vinaceous spotted sheaths, membranaceous near leaf abscission, articulate and leaf-bearing above. ***Leaves*** 5.5–7.2 × 0.38–0.4 cm, linear, sessile, distichous, fleshy, semi-terete, descending, facing downwards, sulcate below, apex obtuse; sheaths 2.5–5.0 cm long, tubular, appressed, green with vinaceous spots as well as leaves. ***Inflorescence*** 7 cm long, apical, pendulous, 1–2-flowered, covered to mid portion by 3 successive, imbricating sheaths; peduncle ca. 4.4 cm long, terete, green with pale vinaceous spots; rachis 2 cm long, terete; sheaths 0.8–2.2 × 0.2–0.3 cm, green with vinaceous spots, ensiform, tubular, laterally compressed, ancipitous, apex acute, decreasing in size. ***Floral bracts*** 2.5 × 1.5 mm, fawn-colored with vinaceous spots, longitudinally triangular, minute, apex acuminate, base truncate. ***Ovary*** ca. 2 cm long, pedicellate, green with vinaceous spots, progressively thickened towards apex, slightly recurved, with 3 longitudinal furrows, one adaxially and two laterally, forming a ventral gibbose vesicle at the apex with basal portion of column. ***Flowers*** 1–2, lax, pendulous, resupinate, unscented; sepals and petals green, dorsally tinged reddish brown, abaxially with vinaceous spots and white margin; lip cream, becoming amber as it ages; column light green with vinaceous dorsal side. ***Dorsal sepal*** 2.4 × 0.4 cm, free, oblong-oblanceolate, arched forward, 5–veined, slightly concave in middle portion towards apex, 3–canaliculate abaxially, margins attenuate, apex obtuse. ***Lateral sepals*** 2.3 × 0.75 cm, free, obliquely oblong-oblanceolate, arched forward, 5-veined, slightly convex at base towards middle portion, margin slightly revolute, involute in middle portion towards apex, apex acute, dorsally keeled. ***Petals*** 2.2 × 0.2 cm, free, linear, slightly incurved, slender, 1–veined, longitudinally somewhat oblique, obtuse, margins slightly recurved. ***Lip*** 2.2 × 2.0 cm, 3–lobed, transversely cordate, apex emarginate, fleshy, rigid, strongly revolute in natural position, margin entire; lateral lobes 11.9 × 7.2 mm, semiorbicular when expanded; mid-lobe 15 × 7.5 mm, bilobate, ecallose, disc with 5 central, parallel, prominent, fleshy and thickened ribs, fused at base, disappearing in the middle of mid-lobe, the two most lateral ribs are much less prominent. ***Column*** 13 × 4 mm, clavate, forming a prominent, ventral, gibbose vesicle at base with apical portion of ovary; clinandrium-hood much reduced, margin entire. ***Anther*** 2.5 × 2.1 mm, broadly elliptical, yellowish green. ***Pollinia*** 4, fulvous, in 2 nearly equal pairs, obovoid, flattened at interfaces, caudicles attaching them in pairs, granulose, viscarium semi-liquid, translucent. **Capsules** not seen.

**Figure 2.**
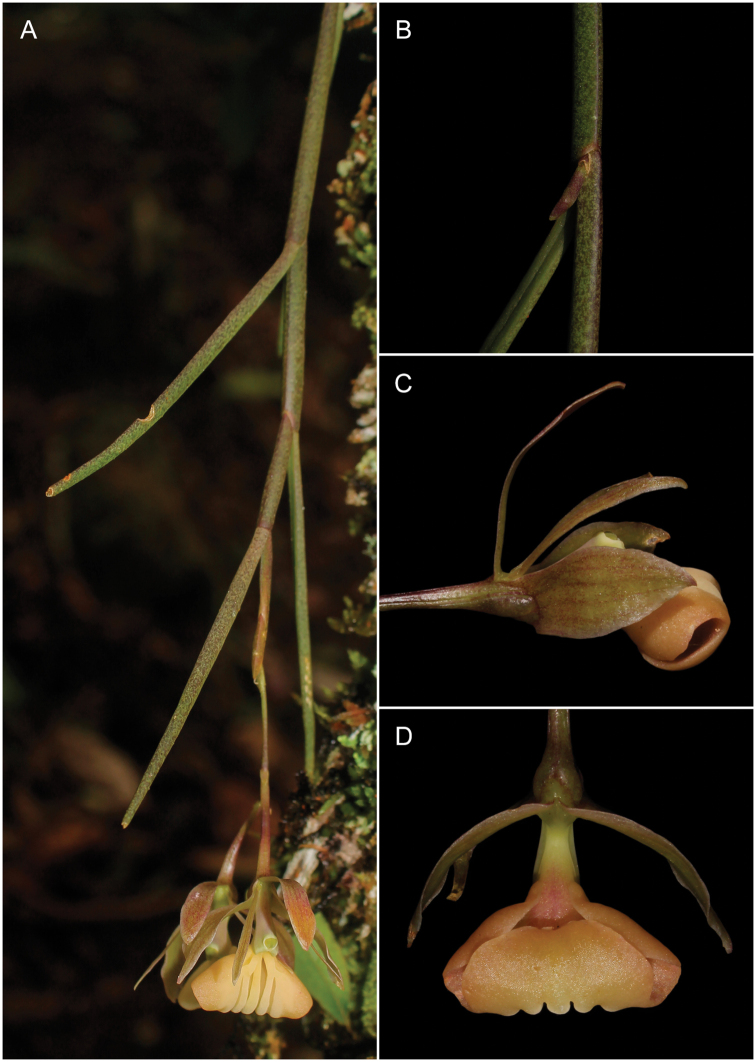
*Epidendrumlufinorum* sp. nov., photographed in situ at the type locality **A** habit with stem apex including inflorescence **B** close-up of a stem segment with a new growth in early stage **C** lateral view of flower **D** abaxial view of flower with ovary and apical vesicle. Photographs by L. Ocupa (based on the holotype: *L. Ocupa* 211).

#### Eponymy.

The epithet is an acronym formed by the first two letters of the names Luis (1966–), Fiorella (1993–) and Noemí (1970–), parents and sister of the first author, to whom he wishes to dedicate this species.

#### Distribution, habitat and comments on the conservation of the species.

**Figure 3.**
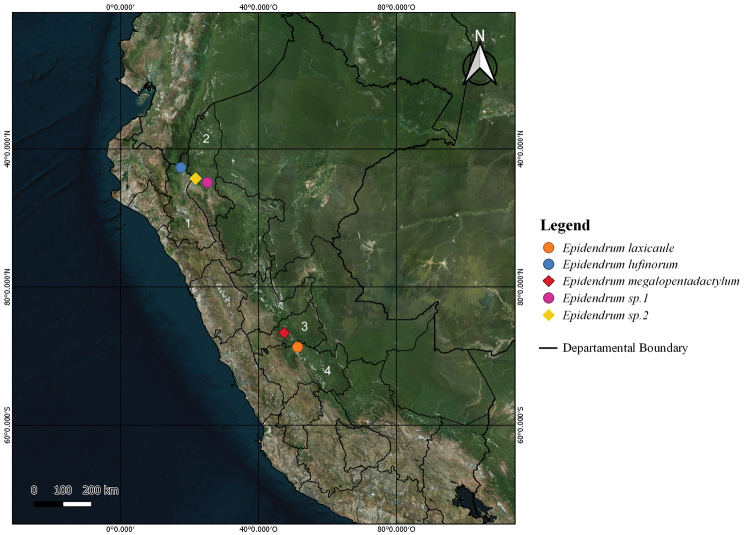
Species distribution map of the *Laxicaule* Group. Regions: **1** Cajamarca **2** Amazonas **3** Pasco **4** Junin Prepared by Luis Ocupa.

This species is currently known only from the type locality in the northern zone of Peru, near the base of the hill known locally as Cerro Parcos, in the village of Villa Rica, San José de Lourdes district (Fig. 3). The habitat of *Epidendrumlufinorum* is within an area with high agricultural activity such as the cultivation of *Coffeaarabica* L. (Rubiaceae Juss.). No more individuals of this species were found in the surrounding areas, but a fertile individual (holotype) and some other small specimens growing as epiphytes on the trunks of *C.arabica* plants, occasionally sharing the same phorophyte with other orchid species such as *Gongoraaromatica* Rchb.f., *Masdevalliaglandulosa* Königer, *Steniacalceolaris* (Garay) Dodson & D.E.Benn. and *Telipogonastroglossus* Rchb.f., which would support the hypothesis of a possible adaptation of this species to anthropized environments. However, its habitat continues to be fragmented as a consequence of tree felling, the expansion of agricultural crops and practices such as pruning and clearing coffee trees of epiphytic plants. The latter is a very common practice among the local population, as a way to maintain optimal conditions for the efficient production of coffee beans, however, it threatens the presence of the few individuals of *E.lufinorum* that may be growing.

Most of the coffee crops were located near secondary forest patches with some individuals of trees as *Cedrelaodorata* L., *Delostomaintegrifolium* D.Don, *Erythrinaedulis* Triana ex Micheli and *Vochysiavismiifolia* Spruce ex Warm.

#### Phenology.

*Epidendrumlufinorum* was observed flowering in April, at the end of the rainy season in that region.

#### Notes on Laxicaule Group.

The combination of a monopodial pendulous habit, linear leaves, an apical inflorescence with few flowers, and a lip with a prominent ribbed disc places the new species in the informal Laxicaule Group.

**Figure 4.**
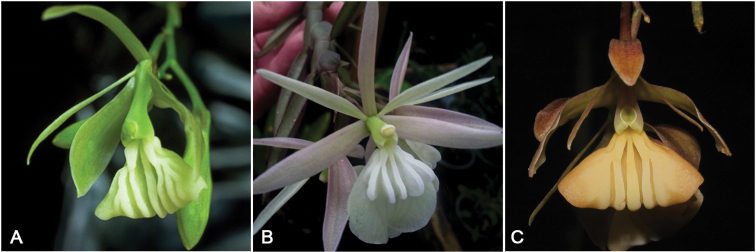
Comparison and flowers close-up of the *Laxicaule Group* species **A***E.laxicaule* (Photo by I. Rolando) **B***E.megalopentadactylum* (Photo by F. Corcuera) **C***E.lufinorum* sp. nov. (Photo by L. Ocupa).

There are two species in the group, both presently known from Peru: *Epidendrumlaxicaule* and *E.megalopentadactylum* (Hágsater and Huayta 2018) reported in Junin and Pasco, respectively (Fig. 4A, B).

Among the two species belonging to the Laxicaule group, *Epidendrumlufinorum* is most similar to *E.laxicaule*, from which it differs in the shape and length of the dorsal sepal; the shape of the petals and the lip, disc ribs position of the lip; shape of the vesicle formed between the ovary and the column as well as the section of the stem. *E.lufinorum* is easily distinguished from the other species of the Laxicaule group by the characteristics indicated in the **Table 1**. It is worth mentioning that the type specimen of *E.lufinorum* we collected is a plant consisting of a flowering primary stem, with a new secondary branch clearly visible in its early stage of development (Fig. 2B).

**Table 1.** Features distinguishing the species of Laxicaule Group.

Character	*E.laxicaule*	*E.megalopentadactylum*	*E.lufinorum*
Stem section	Elliptic	Ancipitose	Terete
Leaf size	8.5 × 0.45–0.47 cm	4.5–7.5 × 1.5–2.5 cm	5–7.2 × 0.38–0.4 cm
Inflorescence	6 cm long	Sessile, compact	7 cm long
Leaf shape	Linear	Narrowly lanceolate	Linear
Cross section leaf	Semiterete	Flat, conduplicate at base	Semiterete
Ovary length	1.3–1.5 cm	1.6 cm	2.0 cm
Ovary vesicle	Globose	Absent	Gibbose
Number of flowers	3–4	3–5	1–2
Dorsal sepal size	3.2 × 0.6 cm	3.8 × 1.0 cm	2.4 × 0.4 cm
Dorsal sepal shape	Narrowly oblanceolate	Narrowly elliptic-ovate	Oblong-oblanceolate
Dorsal sepal apex	Acute	Acute	Obtuse
Lateral sepal size	2.6 × 0.9 cm	4.0 × 1.2 cm	2.3 × 0.75 cm
Lateral sepal shape	Obliquely oblong-oblanceolate	Narrowly elliptic-ovate, slightly oblique	Obliquely oblong-oblanceolate
Petals size	2.8 × 0.2 cm	3.8 × 1.0 cm	2.2 × 0.2 cm
Petals shape	Narrowly linear-lanceolate	Narrowly elliptic	Linear
Petals ápex	Acuminate	Acute	Obtuse
Lip size	2.0 × 2.6 cm	3.3 × 3.1 cm	2.2 × 2.0 cm
Lip shape and lobes	Transverse, 3-lobed	Suborbicular, entire	Transversely cordate, 3-lobed
Disc of lip	5-parallel ribs projecting distally	5-ribs	5-parallel and central ribs
Column size	1.25 cm long	1.0 cm long	1.3 cm long
Column shape	Clavate	Straight	Clavate

**Figure 5.**
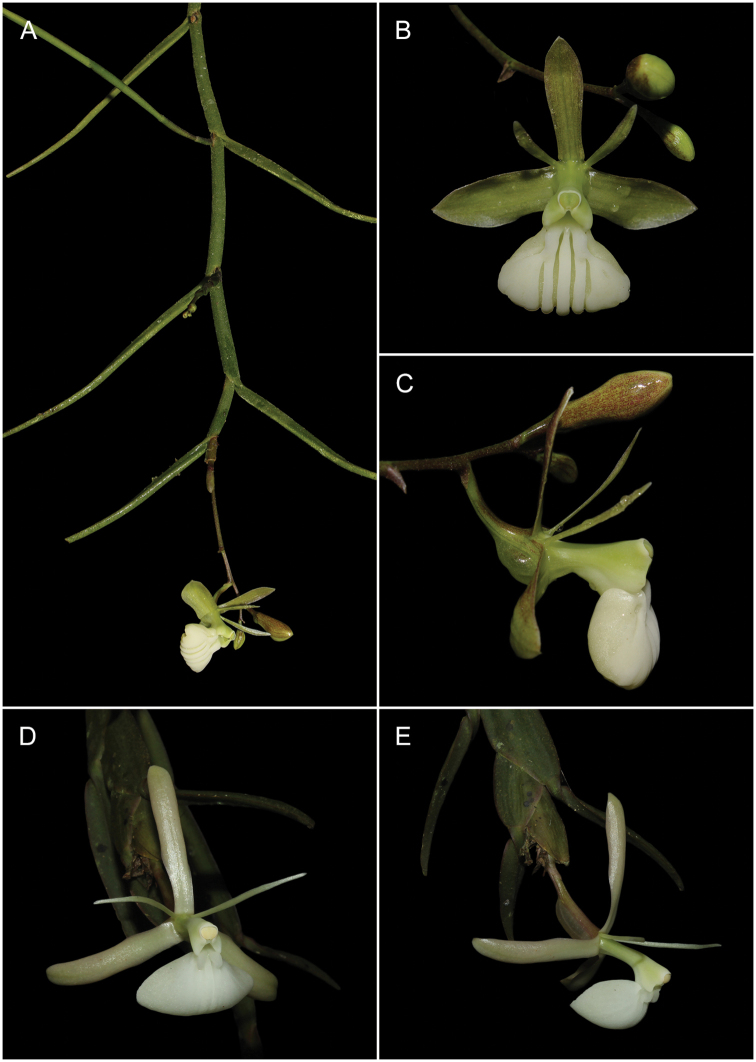
Other undescribed species of the Laxicaule Group found in Amazonas **A, B, C***Epidendrumaff.laxicaule* sp. nov. and E.*lufinorum***D, E***Epidendrumaff.megalopentadactylum* sp. nov. Photographs by L. Pillaca (**A, B, C**) and L. Ocupa (**D, E**).

In May 2015, a specimen of another species, Epidendrumaff.laxicaule sp. nov., was found growing as an epiphyte in a montane forest in the western part of Amazonas department in northern Peru, in the Cajaruro district of Utcubamba province, at an elevation of 1685 m. It was observed and validated with photographs (Fig. 5A, B, C) by Luis Pillaca and shows vegetative and morphological characteristics similar to those of *E.laxicaule* and *E.lufinorum*. However, it has flat and thick, narrow leaves, a much more prominent vesicle at the apex of the ovary and the narrower lip with three parallel and central ribs. Pillaca indicates that the area where the specimen was found has been destroyed due to agricultural encroachment.

A year later, in the same department, but in the province of Bagua, district of Aramango, a specimen of an additional species, Epidendrumaff.megalopentadactylum sp. nov., was found as part of the private collection of Ricardo Saens Saavedra†. This specimen observed and photographed (Fig. 5 D, E) by Luis Ocupa, presents morphological characteristics similar to *E.megalopentadactylum*, such as pendulous plants with flat leaves, conduplicate at the base, strongly laterally compressed and ancipitous stems and an entire lip, but is distinguished by the presence of a vesicle and the lip without ribs.

## Supplementary Material

XML Treatment for Epidendrum lufinorum

## Supplementary materials

Supplementary material 1 Data type species data Explanation note *pidendrumlufinorum*, found in northern Peru, it similar with *Epidendrumlaxicaule* D.E.Benn & Christenson but differs in the shape and length of the dorsal sepal; the shape of the petals and the lip, ribs position of the lip, shape of the vesicle formed between the ovary and the column as well as the section of the stem. https://binary.pensoft.net/file/605552This dataset is made available under the Open Database License (http://opendatacommons.org/licenses/odbl/1.0/). The Open Database License (ODbL) is a license agreement intended to allow users to freely share, modify, and use this Dataset while maintaining this same freedom for others, provided that the original source and author(s) are credited.Luis Ocupa Horna, Eric Hágsater, Marco M. Jiménez
