# Supplementary material for: A new species of Epidendrum L. (Orchidaceae) of pendulous habit from Peru
Source: PhytoKeys. 2021 Nov 3;184:55–66. doi: 10.3897/phytokeys.184.70844 (PMC8580958; doi:10.3897/phytokeys.184.70844)
Supplement: Supplementary material 1 [file phytokeys-184-055-s001.pdf]

A new species of *Epidendrum* (Orchidaceae) of pendulous habit from Peru

Luis Ocupa Horna[1,2], Eric Hágsater[3] & Marco M. Jiménez[4]

1*Departamento de Orquideología, Centro de Investigación en Biología Tropical y Conservación–CINBIOTYC, Piura, Perú.* ***2****Departamento de Orquideología, Instituto de Ciencias Antonio Brack, Lima, Perú.* ***3****Herbario AMO, Montañas Calizas 490, México, CDMX 11000, México.* ***4****Vivero de Conservación La Paphinia, Avenida del Ejército y Juan Izquierdo, Zamora, Zamora Chinchipe, 190102, Ecuador.*

Corresponding author: *Luis Ocupa Horna* (luisocupa.horna@gmail.com)

Abstract
A new species of *Epidendrum*, found in northern Peru, is illustrated with drawings, color photographs and compared with similar species. This species shares morphological characteristics with *Epidendrum laxicaule* D.E. Benn & Christenson but differs in shape and length of the dorsal sepal; shape of the petals and in the position and number of the parallel ribs in the lip.

Keywords
Cajamarca, endemic, *Laxicaule Group*, neotropic, Northern Perú, San Ignacio

Introduction

The genus *Epidendrum* L. (1763: 1347) includes representatives with a diversity of growth habits such as terrestrial, epiphytic and lithophytic plants that occur in different types of vegetation ranging from tropical forests, coastal dunes and scrubs to Andean paramos (Hágsater and Soto 2005, Chase et al. 2015). The genus is to be among the most diverse in the New World orchids, with around 2400 species (Hágsater et al. 2016), 1500 of them have been recently treated and illustrated in the Icones Orchidacearum series (Hágsater and Salazar 1993, Hágsater et. al. 1999, Hágsater and Sánchez 2001, Hágsater and Sánchez 2004, Hágsater and Sánchez 2006, Hágsater and Sánchez 2007, Hágsater and Sánchez 2008, Hágsater and Sánchez 2009, Hágsater and Sánchez 2010, Hágsater and Sánchez 2013, Hágsater and Sánchez 2015, Hágsater and Santiago 2018a, Hágsater and Santiago 2018b, Hágsater and Santiago 2019, Hágsater and Santiago 2020a, Hágsater and Santiago 2020b).

Though there have been many attempts to separate *Epidendrum* into various genera (Hágsater 1985, Hágsater and Soto 2005), the authors based on molecular studies have shown that it is monophyletic and would be best kept as a single genus (Hágsater and Soto 2005, Hágsater et al. 2019, Granados et al. 2020). They also proposed that groups of species within *Epidendrum* can be cohesively aggregated based on vegetative and floral characteristics, which are also supported by molecular information. As this is work in progress, no formal sub-generic classification has been proposed, using informal groups in the process.

This is the case of the Laxicaule group, which is characterized by the pendulous, monopodial plants, with sub-apical branching, the laterally compressed to ancipitose stems, the relatively short leaves, the short racemose, few-flowered inflorescences, the large flowers and the lip with prominent ribs (Hágsater and Huayta 2018). Presently, there are only two species in the group: *Epidendrum laxicaule* D.E. Benn & Christenson and *E. megalopentadactylum* Hágsater & Huayta (Hágsater and Huayta 2018). After studying the material collected in northern Peru, here we describe a new species of *Epidendrum*, with characteristics similar to those found in the Laxicaule Group.

Material & methods

A living plant in flower of the new species was collected in March 2016 during a botanical expedition to Cerro Parcos in the department of Cajamarca, northeastern Peru. The photographs were taken in situ using a Canon® Rebel T3 camera equipped with a Canon EF-S 18-55mm f/3.5-5.6 lens that were used later for preparing the linear drawing and figures. Fresh flowers were preserved in 70% ethanol and 1% glycerol. The specimen collected was dried to make an herbarium specimen, which was afterwards deposited in the Herbarium Truxillense (HUT, acronym following Thiers 2019), Trujillo, Peru.

In order to determine the taxonomic status of the collected specimen, the deposited *Epidendrum* herbarium specimens were examined from USM (acronym following Thiers 2019). The original descriptions from holotypes of related species (Bennett and Christenson 1998, Hágsater and Santiago 2018) were consulted and compared. Additionally, some online resources were accessed such as the JSTOR Global Plants web portal (https://plants.jstor.org). A distribution map of the proposed new species and

morphologically similar species, was prepared using the software QGIS 3.10 (QGIS Development Team 2020). The holotype specimen was collected under the research permit for the project "Estudios Taxonómicos Selectos de la Flora del Norte del Perú" with Resolution N° 247 –2016 –SERFOR/DGGSPFFS and the extension Resol. 430–2017.

Taxonomic treatment

Epidendrum lufinorum **Ocupa & Hágsater, sp. nov.**
Figs 1, 2

Type. PERU. Cajamarca: San Ignacio, in a coffee plantation, close to the caserío Villa Rica, DDM 5° 5.1607’ S, 78° 53.2076’ W, elev. 1690 m, 03 April 2016, *Ocupa 211* (holotype: HUT!).

Diagnosis. *Epidendrum lufinorum* is similar to *E. laxicaule* D.E.Benn & Christenson, but differs in having smaller dorsal sepal 2.4 × 0.4 cm (vs. 3.2 × 0.6 cm) which is oblong-oblanceolate (vs. narrowly oblanceolate), with an obtuse apex (vs. acute), the linear and obtuse petals (vs. narrowly linear-lanceolate and acuminate), and the lip with 3 parallel and central ribs (vs. 5 parallel ribs).

Description. Epiphytic ***herb***, monopodial, branching, pendulous, slender, about 72 cm long including inflorescence. ***Roots*** 2 mm in diameter, basal, white, fleshy,. ***Stems*** ca. 65 cm long, subterete in cross section, ancipitose, new growths produced from the sub-apical nodes of the primary stem, enveloped by tubular, fleshy, articulated, green with vinaceous spotted sheaths, membranaceous near the leaf abscission, leaf-bearing above. ***Leaves*** distichous, fleshy, articulate, descending; sheaths 2.5-5.0 cm long, tubular, appressed, green with vinaceous spots; blade 5.5–7.2 × 0.38–0.4 cm, linear, apex obtuse, facing downwards, sessile, semiterete, furrowed above, , semi-circular in cross section, indented in the flat side, abaxially convex, green with vinaceous spots, . ***Inflorescence*** 7 cm long, apical, pendulous, 1–2-flowered, covered to the mid portion by 3 successive, imbricating sheaths; peduncle ca. 4.4 cm long, terete, green with pale vinaceous spots; sheaths 0.8–2.2 × 0.2–0.3 cm, green with vinaceous spots, ensiform, tubular, laterally compressed, ancipitose, apex acute, decreasing in size. ***Floral bracts*** 2.5 × 1.5 mm, fawn with vinaceus spots, longitudinally triangular, minute, apex acuminate, base truncate. ***Ovary*** ca. 2 cm long, green with vinaceous spots, progressively thickened towards the apex, slightly recurved, with 3 longitudinal furrows, one adaxially and two laterally, forming a ventral gibbose vesicle at the apex with the basal portion of the column. ***Flowers*** 1–2, lax, pendulous, resupinate; sepals and petals green, dorsally tinged reddish brown, abaxially with vinaceous spots and white margin; lip cream, becoming amber as it ages; column light green with vinaceous dorsal side; clinandrium-hood white, anther yellowish green; pollinia fulvous. ***Dorsal sepal*** 2.4 × 0.4 cm, free, oblong-oblanceolate, obtuse, arched forward, 5–veined, slightly concave in the middle portion towards the apex, 3–canalicute abaxially, margins attenuate.. ***Lateral sepals*** 2.3 × 0.75 cm, free, obliquely oblong-oblanceolate, arched forward, 5-veined, slightly convex at the base towards the middle portion, margin slightly revolute, involute in the middle portion towards the apex, apex keeled, acute. ***Petals*** 2.2 × 0.2 cm, free, linear, slightly incurved, slender, 1–veined, longitudinally somewhat oblique, obtuse, , margins slightly recurved, . ***Lip*** 2.2 × 2.0 cm, 3–lobed,

transversely cordate, apex emarginate, fleshy, rigid, strongly revolute in natural position, margin entire, ; lateral lobes suborbicular when expanded; mid-lobe bilobate, , 3 parallel promiente, fleshy thickened central ribs, fused at the base, disappearing in the middle of the mid-lobe, flanked by another lower rib on each side. ***Column*** 13 × 4 mm, clavate, forming a prominent gibbose vesicle at the base with the apical portion of the ovary; clinandrium-hood 2.5 × 2.1 mm, transversally sub-globose, hood reduced, margin entire. ***Anther*** 2.3 × 1.9 mm, broadly elliptical. ***Pollinia*** 4, in 2 nearly equal pairs, obovoid, flattened at the interfaces, caudicles attaching them in pairs, granulose, viscarium semi-liquid, translucent.

Distribution and ecology. This species is currently known only from the type locality in the northern zone of Peru, near the base of the hill known locally as Cerro Parcos, in the village of Villarica, San José de Lourdes district. It grows as an epiphyte on coffee stems (*Coffea arabica*), usually sharing the same phorophyte along with other orchid species such as *Stenia calceolaris* (Garay) Dodson & D.E.Benn., *Masdevallia glandulosa* Königer, *Gongora aromatica* Rchb.f. and *Telipogon astroglossus* Rchb.f. Most of the coffee crops were located near secondary forest patches with some individuals of trees as *Cedrela odorata* L., *Delostoma integrifolium* D.Don, *Erythrina edulis* Triana ex Micheli and *Vochysia vismiifolia* Spruce ex Warm. *Epidendrum lufinorum* was observed flowering in April, at the end of the rainy season in that region (Fig. 3).

Eponymy. The epithet is an acronym formed by the first two letters of the names Luis (1966–), Noemí (1970–) and Fiorella (1993–), parents and sister of the first author, to whom he wishes to dedicate this species.

Preliminary conservation status. *Epidendrum lufinorum* is found in an area of significant human activity and is threatened by agricultural practices such as the trimming and maintenance of coffee bushes. It is a very common practice among the local population, as a way of maintaining optimal conditions for efficient coffee bean production. Another further threat to the species is tree logging caused by the expansion of agriculture.

In 2016, another specimen of Epidendrum has also been found growing as an epiphyte in the mountain forest of the western part of the department of Amazonas in northern Peru, in the Cajaruro district of the Utcubamba Province, at an elevation of 1685 m. It was observed and vouchered with photographs (Fig. 4) by Luis Pillaca and shows a variation in the flat, thick leaves, green and white color of the flowers, the much more prominent vesicle at the apex of the ovary and the apparently narrower lip but with the three parallel and central ribs. L. Pillaca indicates the area where the specimen was found has been now destroyed to create farming fields.

Discussion. By the vegetative architecture of this pendulous species with a primary stem producing shorter branches from sub-apical internodes, peculiar compact, linear leaves and the inflorescence of few flowers without calli and with prominent ribs on the disc of the lip, this species is related to the informal Laxicaule group. There are two species in the group, both presently known from Peru: *Epidendrum laxicaule* and *E. megalopentadactylum* (Hágsater and Huayta 2018) (Fig. 5).

Epidendrum megalopentadactylum, described from central Peru, is recognized by the strongly ancipitose pendant stems, the reclining, narrowly lanceolate leaves, the large, star-shaped, white flowers, the slightly tinged with pink sepals, the greenish white petals and the prominently 5-ribbed lip, reminiscent of a closed fist of 5 bulging fingers.

The new species is more closely related both vegetatively and florally to *Epidendrum laxicaule*, sharing the same vegetative features: linear leaves, strongly recurved 3-lobed lip with very prominent ribs on the disc and the constricted column forming at the base, a vesicle with the apex of the pedicellate ovary. However, *E. lufinorum* differs by having smaller oblong-oblanceolate sepals, linear petals, and the 3-lobed lip with 3 parallel ribs. Also, it produces 1-2 flowers with green sepals and petals, tinged with reddish brown dorsally, with vinaceous spots and the margin white adaxially; the lip is cream, becoming amber as it ages. *Epidendrum. laxicaule* has 3 to 4 larger, green flowers, with the ivory white to pale green lip, the 2.6–3.2 cm long, narrowly oblanceolate sepals, the narrowly linear-lanceolate petals and 5 parallel ribs on the lip.

It is worth mentioning that the type specimen collected of *E. lufinorum* was a young plant with the stem unbranched, but a new secondary branch clearly visible in its early stages (Fig. 2B).

Acknowledgements

The first author wishes to Edwal Rimarachin and his family for access to his orchid collection and support during the expedition. To Ivan Tamayo-Cen for making available the description of *Epidendrum laxicaule*. To Carlos Martel for their corrections and suggestions in this manuscript and to Irwing S. Saldaña for preparing the map of the location of the species. To Felix Corcuera for the photograph of *E. megalopentadactylum* and Luis Pillaca for the photographs of another specimen of *E.* sp. from Amazonas. Finally, we are grateful to the editor and the anonymous reviewers for their suggestions on the manuscript.

References

Bennett DE, Christenson EA (1998) New species of Peruvian Orchidaceae V. Lindleyana 13: 42–45.

Chase MW, Cameron KM, Freudenstein JV, Pridgeon AM, Salazar G, van den Berg C, Schuiteman A (2015) An updated classification of Orchidaceae. Botanical Journal of the Linnean Society 177(2): 151–174.

Granados Mendoza C, Jost M, Hágsater E, Magallón S, van den Berg C, Moriarty Lemmon E, Lemmon AR, Salazar GA, Wanke S (2020) Target Nuclear and Off-Target Plastid Hybrid Enrichment Data Inform a Range of Evolutionary Depths in the Orchid Genus *Epidendrum*. Frontiers in Plant Science 10: 1761. https://doi.org/10.3389/fpls.2019.01761

Hágsater E (1985) Towards an understanding of the genus *Epidendrum*. In Proceedings of the Eleventh World Orchid Conference. American Orchid Society, Miami, Florida, 195–201.

Hágsater E, Huayta A (2018) *Epidendrum megalopentadactylum* Hágsater & Huayta. In: Hágsater E, Santiago E (Eds) The Genus *Epidendrum*, Part 12, Icones Orchidacearum 16 (2): 1683.

Hágsater E, Salazar, GA (1993) The genus *Epidendrum*, part 1, A century of new species in *Epidendrum*, Icones Orchidacearum 2: 201–300.

Hágsater E, Sánchez Saldaña L (Eds) (2001) The genus *Epidendrum*, part 3, A third century of new species in *Epidendrum*, Icones Orchidacearum 4: 401–500.

Hágsater E, Sánchez Saldaña L (Eds) (2004) The genus *Epidendrum*, part 4, A fourth century of new species in *Epidendrum,* Icones Orchidacearum 7: 701–800.

Hágsater E, Sánchez Saldaña L (Eds) (2006) The genus *Epidendrum*, part 5, Species new & old in *Epidendrum*, Icones Orchidacearum 8: 801–900.

Hágsater E, Sánchez Saldaña L (Eds) (2007) The genus *Epidendrum*, part 6, Species new & old in *Epidendrum*, Icones Orchidacearum 9: 901–1000.

Hágsater E, Sánchez Saldaña L (Eds) (2008) The genus *Epidendrum*, part 7, Species new & old in *Epidendrum*, Icones Orchidacearum 11: 1101–1200.

Hágsater E, Sánchez Saldaña L (Eds) (2009) The genus *Epidendrum*, part 8, Species new & old in *Epidendrum*, Icones Orchidacearum 12: 1201–1300.

Hágsater E, Sánchez Saldaña L (Eds) (2010) The genus *Epidendrum*, part 9, Species new & old in *Epidendrum*, Icones Orchidacearum 13: 1301–1400.

Hágsater E, Sánchez Saldaña L (Eds) (2013) The genus *Epidendrum*, part 10, Species new & old in *Epidendrum*, Icones Orchidacearum 14: 1401–1500.

Hágsater E, Sánchez Saldaña L (Eds) (2015) The genus *Epidendrum*, part 11, Species new & old in *Epidendrum*, Icones Orchidacearum 15(1): 1501–1568.

Hágsater E, Sánchez Saldaña L (Eds) (2015) The genus *Epidendrum*, part 11, Species new & old in *Epidendrum*, Icones Orchidacearum 15(2): 1569–1600.

Hágsater E, Santiago E (Eds) (2018a) The genus *Epidendrum*, part 12, Species new & old in *Epidendrum*, Icones Orchidacearum 16(1): 1601–1667.

Hágsater E, Santiago E (Eds) (2018b) The genus *Epidendrum*, part 12, Species new & old in *Epidendrum*, Icones Orchidacearum 16(2): 1668–1700.

Hágsater E, Santiago E (Eds) (2019) The genus *Epidendrum*, part 13, Species new & old in *Epidendrum*, Icones Orchidacearum 17(1): 1701–1756.

Hágsater E, Santiago E (Eds) (2020a) The genus *Epidendrum*, part 13, Species new & old in *Epidendrum*, Icones Orchidacearum 17(2): 1757–1800.

Hágsater E, Santiago E (Eds) (2020b) The genus *Epidendrum*, part 14, Species new & old in *Epidendrum*, Icones Orchidacearum 18(1): 1801–1848.

Hágsater E, Soto MA (2005) *Epidendrum*. In: Pridgeon AM, Cribb PJ, Chase MW, Rasmussen FN (Eds) Genera Orchidacearum 4: Epidendroideae, part one. Oxford University Press, Cambridge, 236–251.

Hágsater E, Sánchez Saldaña L, García-Cruz J (Eds) (1999) The genus *Epidendrum*, part 2, A second century of new species in *Epidendrum*, Icones Orchidacearum 3: 301–400.

Hágsater E, Santiago E, Martínez Rodríguez L (2016) *Epidendrum lasiostachyum* (Orchidaceae): a new Colombian species of the *Epidendrum macrostachyum* group. Lankesteriana 16(1): 27–37. https://doi.org/10.15517/lank.v16i1.23621

Hágsater E, Granados Mendoza C, Salazar GA, Quiroga-González S, Magallón S, van den Berg C, Moriarty Lemmon E, Lemmon AR (2019) Phylogenomics of *Epidendrum*: untangling a Neotropical mega-diversification. In: Pridgeon A, Arosemena AR (Eds) Proceedings, 22 World Orchid Conference, Conference Papers/Systematics. Asociación Ecuatoriana de Orquideología, Guayaquil, Ecuador, 249-254.

Linnaeus C (1763) Species Plantarum, ed. 2 (p. 1347). London: Impenis direct. Laurentii Salvii.

QGIS Development Team (2020) QGIS Geographic Information System. Open Source Geospatial Foundation. http://qgis.org [accessed 10.06.2021]

Thiers B (2019) Index Herbariorum: A global directory of public herbaria and associated staff. New York Botanical Garden Virtual Herbarium, The Bronx. http://sweetgum.nybg.org/ih/ [accessed 10.06.2021]

Figure Legends

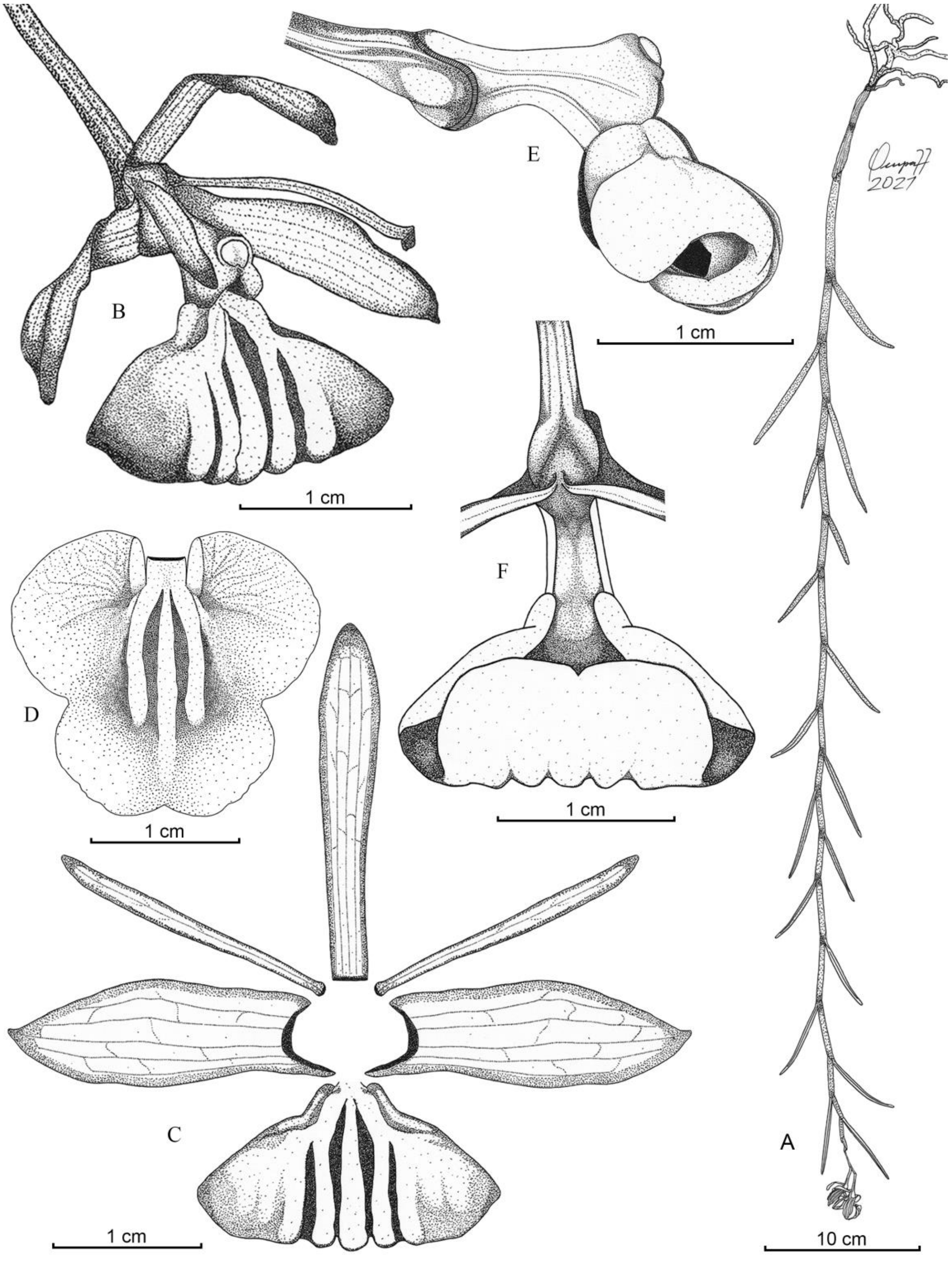

Figure 1. *Epidendrum lufinorum* **A** Habit **B** Flower **C** Dissected perianth. **D** Lip extended **E** Column, lip and ovary, lateral view **F** Lip in natural position, abaxial view. Illustration by L. Ocupa from the holotype (*L. Ocupa* 211, HUT).

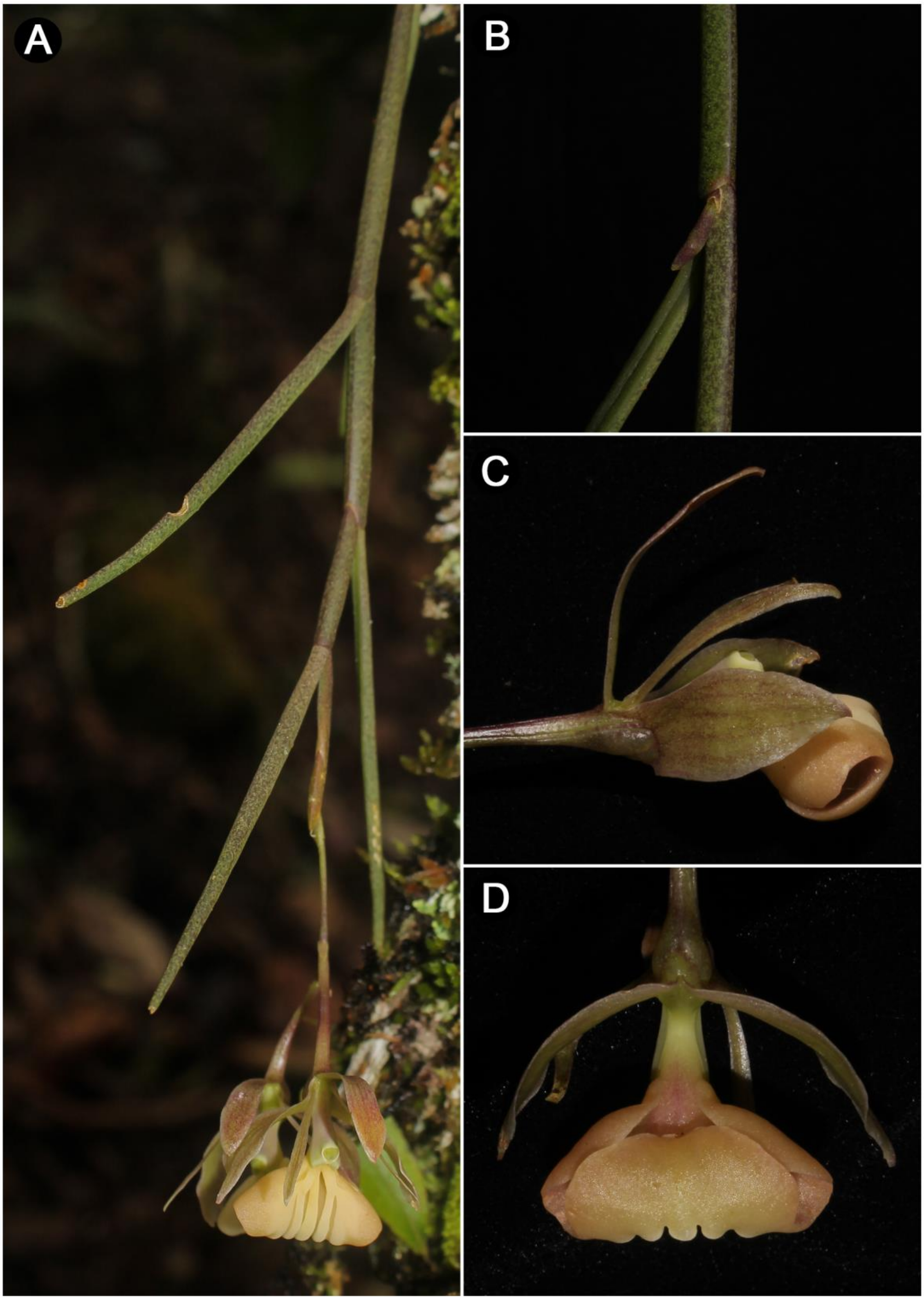

Figure 2. *Epidendrum lufinorum*, photographed in situ at the type locality **A** Stem apex including inflorescence **B** Close-up of a stem segment with a probably new growth in early stage **C** Lateral view of flower **D** Abaxial view of flower with ovary and apical vesicle. Photographs by L. Ocupa (based on the holotype: *L. Ocupa* 211).

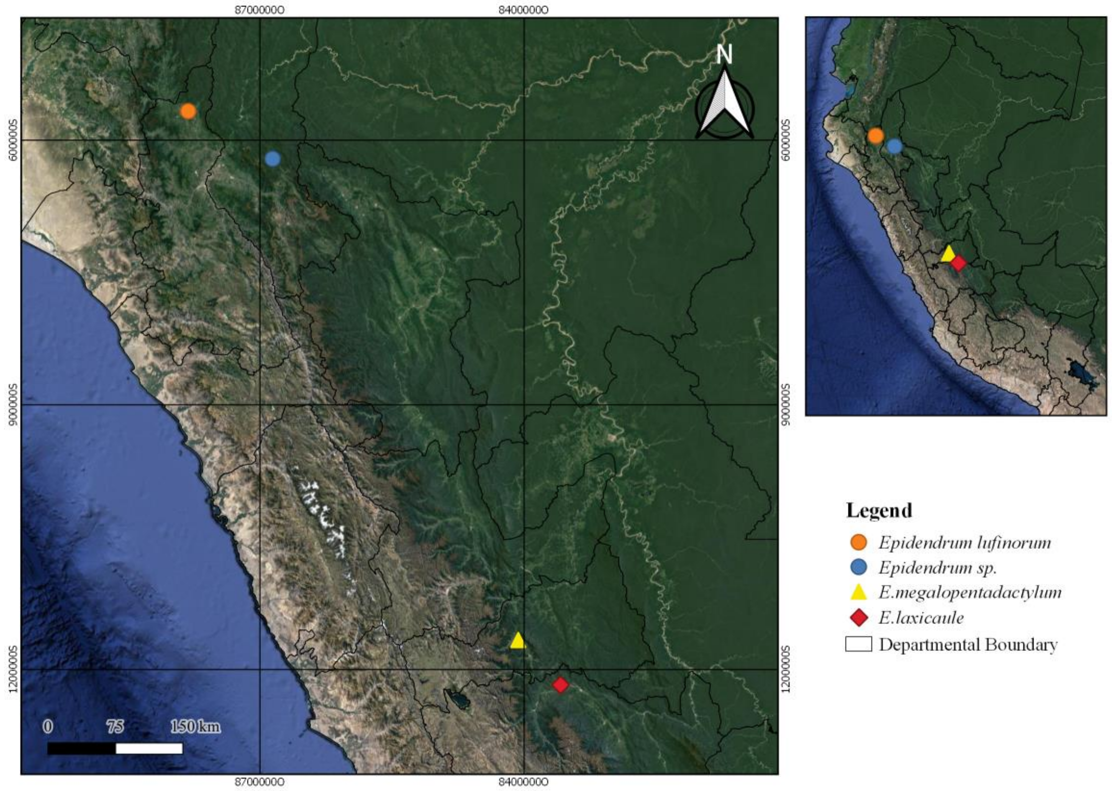

Figure 3. Species distribution map of the *Laxicaule* Group. Circle orange: *E. lufinorum*. Triangle yellow: *E. megalopentadactylum* (based on Hágsater & Huayta 2018). Rhombus red: *E. laxicaule* (based on Bennett & Christenson 1998). Circle blue: *E. sp.* Pillaca (based on Pillaca s.n.). Prepared by Irwing S. Saldaña.

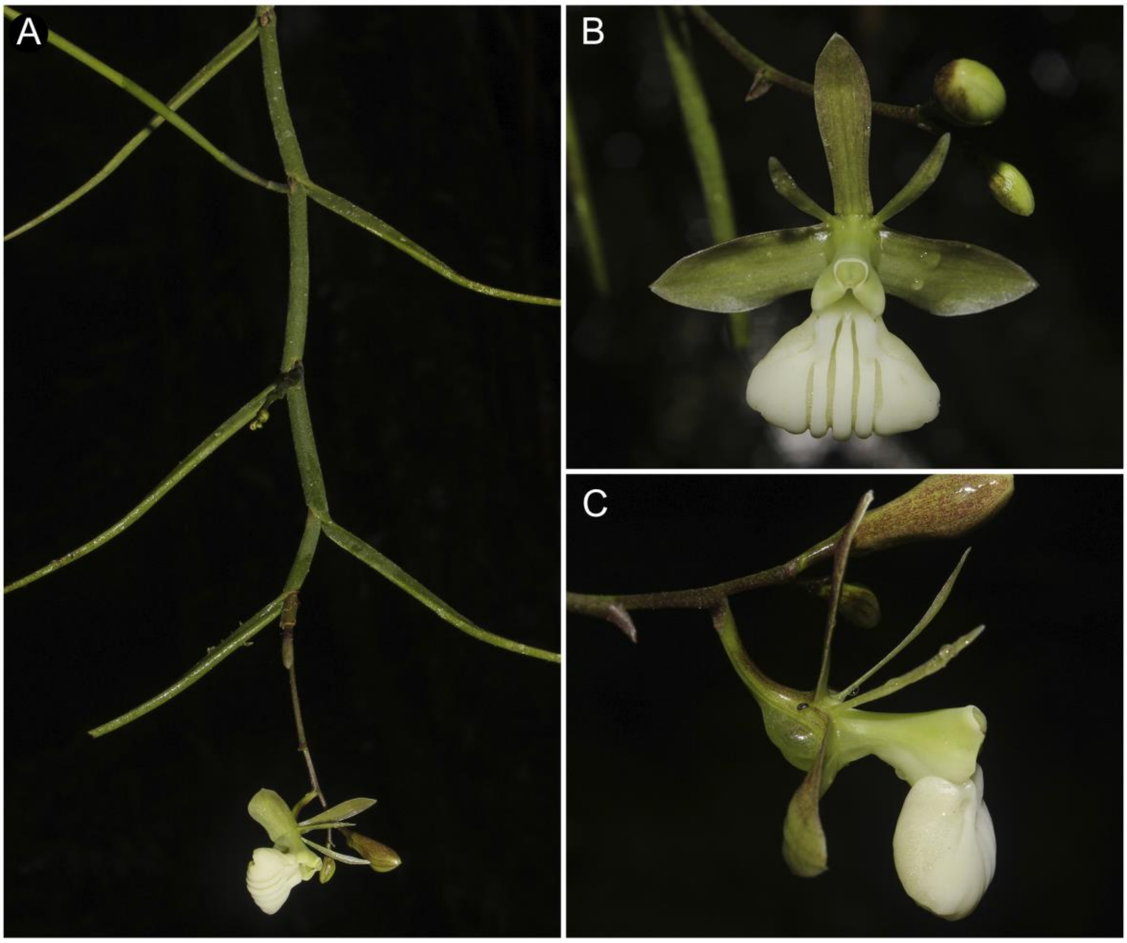

Figure 4. Another specimen of *Epidendrum* sp. reported in the department of Amazonas **A** Apical portion of stem including inflorescence **B** Flower, frontal view **C** Flower, lateral view. Photographs by L. Pillaca.

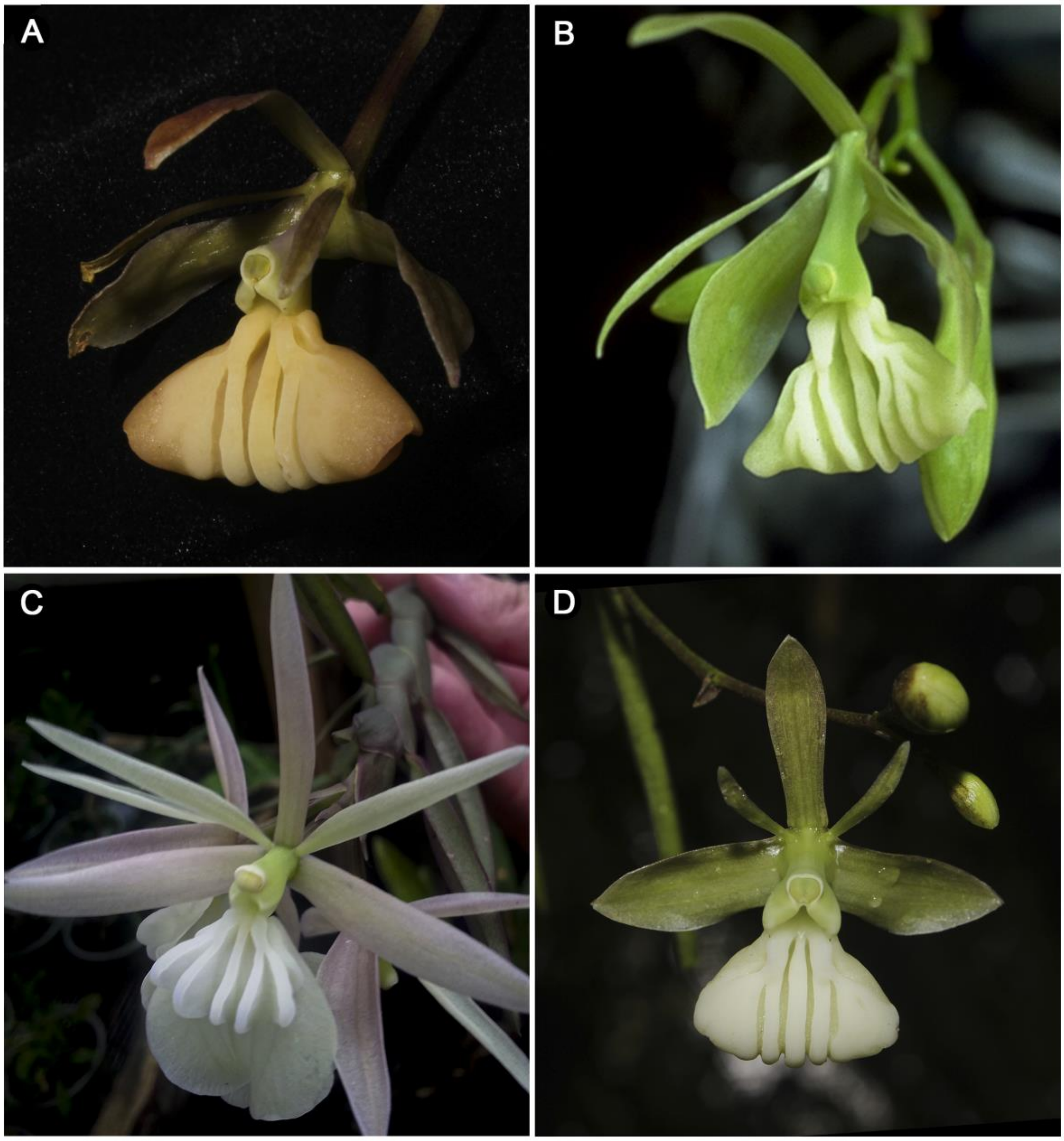

Figure 5. Comparison and flowers close-up of the *Laxicaule group* species: **A** *E. lufinorum* (Photo by L. Ocupa) **B** *E. laxicaule* (Photo by I. Rolando) **C** *E. megalopentadactylum* (Photo by F. Corcuera) **D** *E.* sp. (Photo by L. Pillaca)
